# Pancreatic cyst fluid harbors a unique microbiome

**DOI:** 10.1186/s40168-017-0363-6

**Published:** 2017-11-09

**Authors:** Shan Li, Gwenny M. Fuhler, Nahush BN, Tony Jose, Marco J. Bruno, Maikel P. Peppelenbosch, Sergey R. Konstantinov

**Affiliations:** 1 Department of Gastroenterology and Hepatology, Erasmus MC – University Medical Center Rotterdam, ‘s Gravendijkwal 230, 3015 CE Rotterdam, The Netherlands; 2 Erasmus Medical Center Cancer Institute, Rotterdam, The Netherlands; 3 Clevergene Biocorp Private Limited, Bangalore, India; 4 Janssen Vaccines and Prevention B.V., Leiden, The Netherlands

**Keywords:** 16S rRNA gene, Pancreatic cyst fluid, Cystic tumors, *Fusobacterium* spp., Bacterial translocation, NGS, Human Microbiome Project

## Abstract

**Background:**

It is clear that specific intestinal bacteria are involved in the development of different premalignant conditions along the gastrointestinal tract. An analysis of the microbial constituents in the context of pancreatic cystic lesions has, however, as yet not been performed. This consideration prompted us to explore whether endoscopically obtained pancreatic cyst fluids (PCF) contain bacterial DNA and to determine the genera of bacteria present in such material.

**Methods:**

Total DNA was isolated from 69 PCF samples. Bacterial 16S rRNA gene-specific PCR was performed followed by Sanger sequencing and de novo deep sequencing for the V3-V4 variable region of 16S rRNA gene.

**Results:**

We observed that 98.2% of the samples were positive in conventional PCR, and that 100% of selected PCF samples (*n* = 33) were positive for bacterial microbiota as determined by next generation sequencing (NGS). Comprehensive NGS data analysis of PCF showed the presence of 408 genera of bacteria, of which 17 bacterial genera were uniquely abundant to PCF, when compared to the Human Microbiome Project (HMP) database and 15 bacterial microbiota were uniquely abundant in HMP only. *Bacteroides* spp., *Escherichia/Shigella* spp., and *Acidaminococcus* spp. which were predominant in PCF, while also a substantial *Staphylococcus* spp. and *Fusobacterium* spp*.* component was detected.

**Conclusion:**

These results reveal and characterize an apparently specific bacterial ecosystem in pancreatic cyst fluid samples and may reflect the local microbiota in the pancreas. Some taxa with potential deleterious functions are present in the bacterial abundance profiles, suggesting that the unique microbiome in this specific niche may contribute to neoplastic processes in the pancreas. Further studies are needed to explore the intricate relationship between pathophysiological status in the host pancreas and its microbiota.

**Electronic supplementary material:**

The online version of this article (10.1186/s40168-017-0363-6) contains supplementary material, which is available to authorized users.

## Background

Pancreatic cysts are fluid-filled neoplasms that can be detected with a frequency of up to 2% in the general asymptomatic adult population and have a low, but not negligible risk for evolving into pancreatic ductal adenocarcinoma, while accounting for up to 5% of the total incidence of pancreatic cancerous lesions [1, 2]. The vast majority of cysts are coincidentally found during cross-sectional imaging done for other reasons than cyst-related symptoms. Optimal clinical management of pancreatic cysts remains controversial, but there is consensus in the field that increased insight into the molecular pathogenesis of pancreatic cysts may guide development of rational strategies in this respect. Unfortunately, the etiology of pancreatic cysts remains largely obscure.

Progress with respect to understanding the nature and natural history of pancreatic cysts is compounded by the presence of different types of this lesion. Grutzmann et al. and Farrell et al. have attempted to classify the different types of pancreatic cyst lesions and distinguish among others as intra-ductal papillary mucinous neoplasms (IPMNs), mucinous cystic neoplasms (MCN), serous cystadenomas (SCA), and pseudocysts [3–5]. In general, it is assumed that IPMN and MCN pose a higher risk of developing into carcinoma, with IPMN being more prevalent compared with MCN [1, 6]. IPMNs are further classified as main branch, side branch, or mixed types, based on the involvement of the duct in the pancreas [5]. Presently, there are no validated biomarkers to identify cystic lesions that require surgical resection and this constitutes a major challenge in this field. Although pancreatic lesions develop into malignancy in only up to 3 % of cases, 10 % of patients with such lesions undergo resection [5] suggesting the need for superior clinical tests and patients’ stratification prior surgery. Currently, the decision for resection of pancreatic cyst lesions and/or continued monitoring is made according to the Sendai guidelines after evaluation of different clinical tests [7–9]. The available clinical tests include different biochemical analyses, cytology, pathological assessment of fine needle biopsy or aspiration material, endoscopic ultra-sonography (EUS), and radiological diagnosis such as endoscopic retrograde cholangiopancreatography (ERCP), magnetic resonance cholangiopancreatography (MRCP), and whole-body computerized tomography (CT). The inter-observer agreement, however, between different modalities remains moderate [10]. Therefore, a set of preoperative biochemical analyses have been increasingly used in clinical decision-making. This includes the study of cyst fluids and serum for the characteristic presence of carcinoembryonic antigen (CEA), cancer antigen 19.9 (CA19.9), cancer antigen 72.4 (CA72.4), cancer antigen 15.3 (CA15.3), pancreatic amylase, and mucin antigens, along with other cyst characteristics [11, 12]. Other tests are based on specific analysis of different genetic modalities like K-RAS mutation and integrity, but efforts attempting to provide clinical validation for such tests have largely proven unsuccessful [1, 6, 13, 14]. Increased insight into the factors that facilitate the development of cystic lesions would evidently benefit the identification of tests capable of providing guidance for clinical management of asymptomatic patients exhibiting pancreatic cysts.

Intriguingly, the human gut microbiome has emerged recently as an important environmental factor linked to the development of different intestinal and extra-intestinal malignancies [15–17]. In the stomach, *Helicobacter pylori* remains the archetypical example of a prokaryotic organism that can initiate a cascade of molecular events finally leading to full-blown cancer, whereas in the colon, various organisms and especially *Fusobacteria* have been linked to the appearance of dysplasia (reviewed in [17]). Whether the appearance of pancreatic cysts is linked to the presence of bacteria per se and if so whether specific types of bacteria are associated to the presence of cystic pancreatic neoplasms has remained unexplored. If, however, the presence of pancreatic cyst can be linked to the microbiome, this would entail a significant step forward with respect to our understanding of pancreatic cystogenesis.

The above-mentioned considerations prompted us to explore the potential microbial component of pancreatic cyst fluid. The results show that presence of bacterial DNA is common to such material and that especially *Fusobacterium* spp. and *Bacteroides* spp. are prominently present in such material. As some of these bacterial species have been linked to dysplastic processes elsewhere in the tracts, a causal link between the presence of such bacteria and pancreatic neoplasm may also exist and the results may indicate that bacterial colonization of pancreatic cyst fluid is a regular phenomenon.

## Methods

### Patient samples and pancreatic cyst fluid collection

A cohort of 69 patients with suspected cystic lesions was established between the period of 2008 and 2013 (Table 1 and Additional file 1).The pancreatic cyst fluids (PCF) were collected after a signed informed consent from these patients, who were undergoing endoscopic ultrasound fine needle aspiration (EUS-FNA) at the Department of Gastroenterology, Erasmus MC, The Netherlands. Following collection, pancreatic cyst fluids were transferred to the laboratory and stored at − 150 °C until analysis.

**Table 1:** Characteristics and clinicopathological features of the patients with pancreatic cyst

Patient characteristics		IPMN (*n* = 27)		MCN (*n* = 13)	Others (*n* = 11)		Pseudocysts (*n* = 9)	Serous cystadenoma (*n* = 9)
Types of cysts (%) (*n* = 69)		Main branch IPMN	2.9%		No definite clinical diagnosis (others)	6.0%		
		Mixed type IPMN	5.8%		NET (others)	3.0%		
		Multifocal side branch IPMN	5.8%		Acinar cell carcinoma (others)	1.0%		
		Side branch IPMN	17.0%		Simple cyst (others)	3.0%		
		IPMN	7.2%		Cystic GIST; no communication PD (others)	3.0%		
Total percentage of samples in each pancreatic cyst		39.10%		18.8%	15.90%		13.0%	13.0%
Resected sample percentage		11.6%		13%	4.3%		2.9%	0.0%
Dysplasia	No Dysplasia	66.7%		53.8%	100%		100%	100%
	Adenoma	11.1%		38.5%	0.0%		0.0%	0.0%
	Moderate dysplasia	11.1%		7.7%	0.0%		0.0%	0.0%
	Carcinoma in situ	11.1%		0.0%	0.0%		0.0%	0.0%
Gender	Male	15.9%		0.0%	7.2%		7.2%	2.9%
	Female	21.7%		17.4%	7.2%		5.8%	10.1%
	Not available	1.4%		1.4%	1.4%		0.0%	0.0%
Average age		68 years 11 months 22 days		53 years 7 months 14 days	62 years 5 months 14 days		54 years 8 months 19 days	59 years 9 months 18 days

### DNA isolation

Approximately 300 μl from 69 pancreatic cyst fluid samples were used for total DNA isolation. After bead beating (Fast Prep®-24 Instrument), the supernatant and pellet were separated by centrifugation at 13000 rpm for 1 min and both pellet and supernatant were used for total DNA isolation, using the Wizard DNA isolation kit as specified by the manufacturer’s protocol (catalog no. A1620, Promega BNL B.V, The Netherlands). Isolated DNA was equilibrated in the DNA rehydration solution from the kit and quantified on nanodrop-2000 spectrophotometer (Isogen Life Science BV, De Meern, The Netherlands). Pancreatic cyst fluid DNA was diluted to 1 ng/μl for the PCR analyses and subsequently stored at − 20 °C.

### PCR analyses

**Fig. 1:**
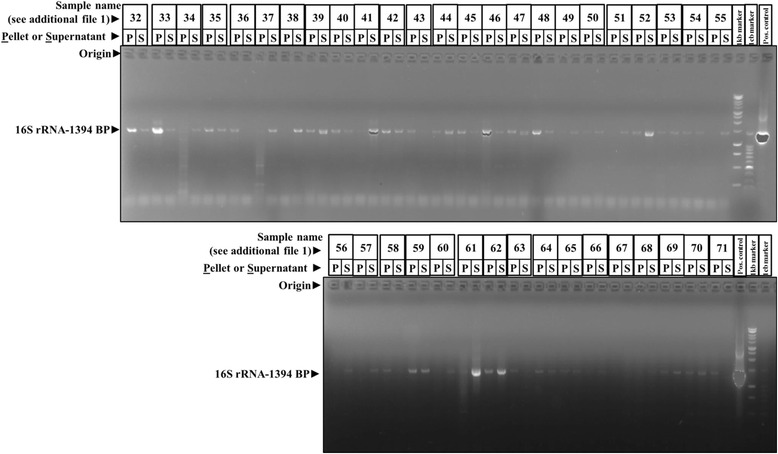
Abundance of bacterial DNA in pancreatic cyst fluid. A series of pancreatic cyst fluids (patient characteristics can be found by linking sample name to the patient information provided through additional Table 1) were exposed to bead beating and centrifugation followed by DNA extraction from both the pellet as well as the supernatant. Subsequently, the DNA was probed for the presence of sequence coding for bacterial 16S RNA through PCR. Reaction products were resolved by gel electrophoresis employing an agarose gel. The results show that bacterial DNA is commonly found in pancreatic cyst fluids

Total DNA isolated from pancreatic cyst fluids was used for bacterial 16S rRNA gene detection using conventional PCR. GoTaq^®^ Flexi DNA polymerase kit (Promega BNL B.V, The Netherlands) and universal 16S rRNA primers were used (Bacteria cPCR-27F 5′-AGAGTTTGATCCTGGCTCAG-3′, Bacteria cPCR-1401R 5′-CGGTGTGTACAAGACCC-3′, 1394 bp product size, PCR condition, 95 °C for 6 min; 30 cycles of 95 °C denaturation for 30s, 50 °C primer annealing for 30 s and 72 °C elongation for 90 s; and final elongation of 7 min at 72 °C). The 16S rRNA gene amplicons were run on 1% agarose gels (Sigma Aldrich, The Netherlands), and the positive samples were selected by the presence of 16S rRNA gene 1394 bp band (Fig. 1).

### 16S rRNA gene sequencing analyses

#### Sanger sequencing

Sanger sequencing was done in order to identify the bacterial 16S rRNA genes present in the PCF. The PCR products generated using universal primers of 16S rRNA were sequenced using F primer 5′-CTTAGGAATGAGACAGAGATG-3′ through LGC Genomics GMBH, Germany. The chromatograms were analyzed, sequences were curated and identified using web databases of integrated microbial genomes and 16S rRNA-specific nucleotide BLAST. The sequences have been deposited at NCBI under gene accession identifiers MF061964–MF061990.

#### Deep sequencing

From this cohort of 69 PCF samples, DNA of 33 samples [(47.8%), IPMN (*n* = 9), MCN (*n* = 7), Pseudocysts (*n* = 8), and SCA (*n* = 9)] was selected for de novo 16S rRNA gene amplicon sequencing by Macrogen (South Korea). 16S rRNA gene amplicon sequencing was performed on the V3-V4 variable region using Illumina Miseq adapter PCR followed by clustering and sequencing. The raw images generated are used by MCS (MiSeq Control Software v2.4.1.3) for system control and base calling through an integrated primary analysis software called RTA (real-time analysis. v1.18.54.0). The BCL binary (base calls) is converted into FASTQ utilizing illumine package MSR (MiSeq Reporter).

#### Deep sequencing data analysis

Two types of deep sequencing analyses were performed, one with paired-end reads alone discarding the single pair reads leading to 408 genera identification, and one with paired-end reads and single pair reads leading to identification of 785 genera [18]. The data generated via paired-end reads alone was further used for downstream analysis for comparison of the PCL bacteria population vs human microbiome project samples of 13 different body sites and comparisons between the types of clinically defined cysts types and resection (Additional file 1).

#### Pre-processing of amplicon reads and sequence data QC

Illumina Miseq sequence data was quality checked using FastQC and Rqc software’s [19]. Base call quality distribution, percentage of bases with quality scores above Q20 and Q30, GC percentage, and sequencing adapter contamination were used for assessing the data quality. The details of the number of reads and base quality obtained for each sample are provided in Additional file 2.

#### Taxonomic classification and OTU clustering

For downstream analysis of the metagenome data, “mothur” software bundle was used [20].The quality-filtered sequence reads were imported into mothur, and the read pairs were aligned with each other to form contigs based on sequence consensus. This results in longer contigs that span the targeted V3-V4 hypervariable region. These contigs were screened and only those between 470 and 500 bp were taken for further analysis. Contigs with ambiguous base calls were rejected; high-quality contigs were checked for identical sequences and duplicates merged.

The filtered contigs were aligned to a known database for 16S rRNA [Silva V.119] [21]. These classified contigs were filtered for any undesired lineage from the taxonomy file. Any ambiguous contigs aligning to untargeted regions [other than V3-V4] were discarded. Using UCHIME algorithm, chimeric contigs were flagged and removed; a known reference for chimeric sequence was cross referenced [22]. These final set of contigs were then phylotype binned into operational taxonomic units (OTU) based on the Silva V.119 database and the abundance of each OTU in the population was estimated.

#### Comparison of the taxonomic classification of the pancreatic microbiomes with those from other body sites

In order to identify microbial signature of pancreatic cysts, a comparative analysis was carried out against organ-specific bacterial profiles obtained from the NIH Human Microbiome Project (HMP) database [23]. The pancreatic microbial profile of the current study was compared with microbial profiles of 13 different body sites downloaded from HMP (SRA numbers provided in Additional file 3). The notion that bacteria in the gut may affect diseases outside the gut and vice versa is gradually accepted, and we therefore considered both anatomical sited in close proximity to the pancreas as well as more distant sites.

The comparative analysis was carried out between anterior nares, antecubital fossa, buccal mucosa, gingiva, hard palate, mid vagina, palatine tonsils, posterior fornix, retroauricular crease, saliva, stool, throat, and tongue. Although phylotype-based analysis is limited as compared to distance-based OTU classification, it allows for investigating the relationship of the PCF microbiota to previously characterized microbes in the HMP database.

Two analyses were performed for identifying the unique microbiota when PCF commensals were compared to the bacterial communities associated with 13 different body sites. First, a pairwise binomial test was carried out against each organ in order to identify significantly (FDR < 0.05, FC > 3) and absolute abundance difference of 10 abundant bacterial species in pancreas. Second, in order to identify the relatedness of the PCF microbiomes with those from other body sites, we compared bacterial communities of the PCF samples to those of taxonomic profiles from 13 other body sites from the HMP database by using principal component analysis (PCA). Third, statistical comparisons of these microbiomes were performed using the STAMP analysis package. The statistical tests used was ANOVA and Welch’s *t* test, with Benjamini-Hochberg multiple test corrections. Those taxa having a higher abundance in the pancreatic cysts (with corrected *P* value < 0.01) were identified as those that are specifically abundant in the PCF.

#### Diversity analysis between the cyst types

Using STAMP statistical analysis package, those taxa having a higher abundance between the test groups IPMN vs pseudocyst vs MCN vs SCA were identified as specific for the groups to be classified based on the bacterial population. The cysts were grouped based on the pathological classifications, and they are tested using White’s non parametric *t* test, two tailed with Benjamini-Hochberg multiple test corrections of *P* value (*P* < 0.05) [24]. The measured levels of CEA and CA19.9 ranges were used for classification of cysts as cysts, benign cysts, and malignant cysts according to study conducted by Talar-Wojnarowska et al. [25]. Then the classified data was used for the specific bacterial taxa identification between the resected types using the same parameters.

#### Statistics and calculations

All the statistical analysis were done using excel, Graphpad Prism 5.0., and STAMP statistical analysis software. When appropriate, results were Bonferroni corrected in graphpad analysis and Benjamini-Hochberg multiple test corrections in STAMP. *P* values < 0.05 (between cysts and resection types) and < 0.01 [between body sites (NGS)] were considered significant.

## Results

### Study sample characteristics

For analysis of the potential microbial component of pancreatic cyst fluid (PCF), material collected from 69 patients was used. The characteristics of these patients are listed in Table 1 and Additional file 1. Of the PCF analyzed, 27 were obtained from patients harboring an IPMN (39.1%), including two patients with main branch IPMN (2.9%), four patients with mixed type IPMN (5.8%), 12 patients with a unifocal side-branch IPMN (17.4%), and four patients presenting with a multifocal side-branch IPMN (5.8%). In five patients, the type of IPMN was unclassified (7.2%). In addition, our cohort contained 13 patients with an MCN (18.8%), nine patients with pseudocysts (13.0%), and nine patients with serous cystadenomas (13.0%). Finally, the cohort contained 11 patients with apparently multiple forms of cystic lesions, gastrointestinal stromal tumor (GIST), neuroendocrine tumors (NET), or having no definitive clinical diagnosis and for this study, these patient were classified as “others” (15.9%). We concluded that this cohort would allow the study of potential microbiological constituents of PCF and to relate results to the clinical phenotype of the patient from which the fluid was obtained.

### Bacterial DNA is commonly present in EUS-FNA-collected pancreatic cyst fluids

As it is as yet unknown whether pancreatic cystic fluid hosts a microbiological component, analyzed the EUS-FNA-collected pancreatic cyst fluid obtained from our cohort for the presence of significant amounts of bacterial DNA. Importantly, we found that the majority of these fluid samples were rich for bacterial DNA, with 16S rRNA PCR demonstrating the presence of bacterial DNA in 64 (92.8%) out of the 69 samples (Table 2 and Fig. 1). The presence of bacterial DNA in cyst fluid did not statistically relate to the type of lesion from which it was obtained (*P* value > 0.99; χ^2^ test): in mucinous cystic neoplasms, 100% of samples contained significant amounts of bacterial DNA; in IPMNs, this number was 92.6%; in pseudocysts, 88.9% of PCF were positive for bacterial DNA; in serous cystadenomas, 88.9%; whereas in the group of others which included GIST, NET, and clinically undefined samples, 90.9% displayed significant amounts of bacterial DNA (Additional file 1 and Table 2).

**Table 2:** Bacterial ecosystems characteristics identified from the pancreatic cyst fluids using PCR, Sanger sequencing, and next-generation sequencing (NGS)

Bacterial ecosystem characteristics						
Patient characteristics		IPMN (*n* = 27)	MCN (*n* = 13)	Others (*n* = 11)	Pseudocysts (*n* = 9)	Serous cystadenoma (*n* = 9)
16S rRNA PCR (universal 16S rRNA gene primers)	Bacteria present	92.6%	100%	90.9%	88.9%	88.9%
	Bacteria absent	7.4%	0.0%	9.1%	11.1%	11.1%
Sanger sequencing	Bacteria detected	*Bacillus* spp. *Fusobacterium* spp., *Orpinomyces* spp. *Anaerococcus* spp., *Caldimonas* spp., *Acinetobacter* spp., *Bacillus* spp*.*	*Fusobacterium* spp., *Bacillus* spp*., Orpinomyces* spp., *Microcystis* spp., *Staphylococcus* spp.	*Fusobacterium* spp.	*Caldimonas* spp., *Propionibacterium* spp., *Fusobacterium* spp., *Curvibacter* spp., *Escherichia* spp., *Bacillus* spp.	*Arthrobacter* spp., *Bacillus* spp., *Bacteroides* spp., *Ruminococcus* spp.
16S rRNA (NGS) (*n* = 33)	Bacteria present	100% (*n* = 9)	100% (*n* = 7)	NA	100% (*n* = 8)	100% (*n* = 9)
Bacteria detected by 16S rRNA gene V3-V4 variable region NSG (*n* = 33)		*Bacteroides—15.45%* *Escherichia/Shigella—9.88%* *Faecalibacterium—8.57%* *Acidaminococcus—5.75%* *Sphingomonas—4.87%* *Others—55.49%*	*Bacteroides—17.06%* *Escherichia/Shigella—10.17%* *Faecalibacterium—6.95%* *Acidaminococcus—5.22%* *Sphingomonas—6.48%* *Others—54.12%*	NA	*Bacteroides—16.59%* *Escherichia/Shigella—10.55%* *Faecalibacterium—6.81%* *Acidaminococcus—6.23%* *Sphingomonas—5.40%* *Others—54.42%*	*Bacteroides—16.73%* *Escherichia/Shigella—9.97%* *Faecalibacterium—6.64%* *Acidaminococcus—6.24%* *Sphingomonas—4.81%* *Others—55.62%*

NA not applicable

### Microbial composition of pancreatic cyst fluid with a differential clinical aspect

The presence of a microbial component in PCF raises obvious questions as to the identity of the organisms apparently present in such fluid. Sanger sequencing of PCR products generated using universal 16S rRNA demonstrated that *Fusobacterium* spp. is present in 13 out of all 69 PCF samples (18.84%). Another predominating bacterium in PCF was *Bacillus* spp. which was present in 16 out of 69 (23.19%) samples. The presence of other bacteria was also noted which included *Ruminococcus* spp., *Staphylococcus* spp., *Caldimonas* spp., *Arthrobacter* spp., *Acinetobacter* spp., *Bacteroides* spp., *Orpinomyces* spp., and *Anaerococcus* spp. (Table 2).

**Fig. 2:**
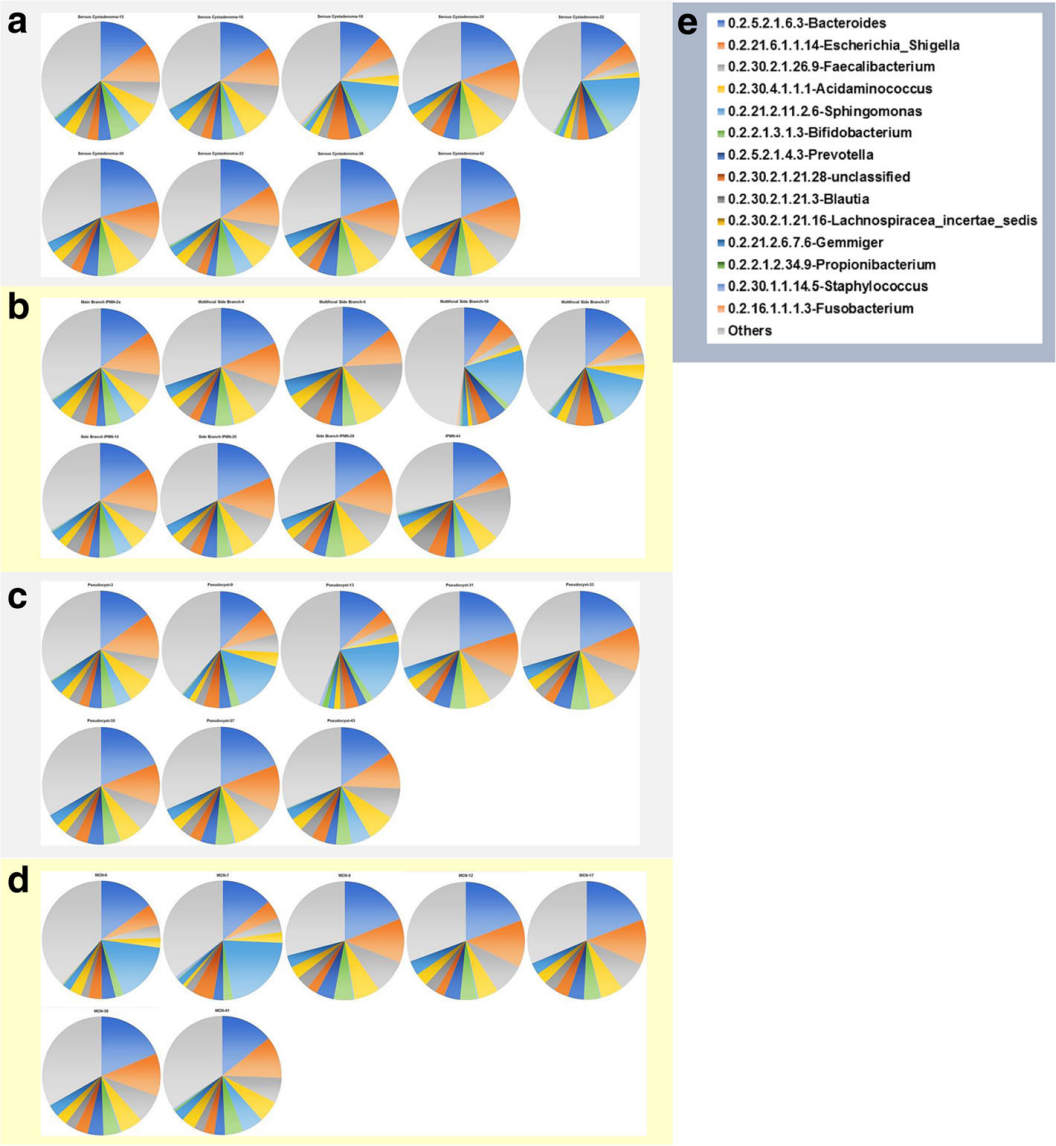
Taxonomic composition of microbiological genera of pancreatic cyst fluid. DNA isolated from 33 pancreatic cyst fluid samples (selected based on strong PCR signals for 16S) was characterized by 16S rRNA NGS for its microbiological composition. The numbers indicated correspond to the sample names in Additional file 1. **a** Serous cystadenoma aspiration fluid microbiome. **b** Intraductal papillary mucinous neoplasm aspiration fluid microbiome. **c** Pseudocyst aspiration fluid microbiome. **d** Mucinous cystic neoplasm aspiration fluid microbiome. **e** Legend of the pie chart

**Fig. 3:**
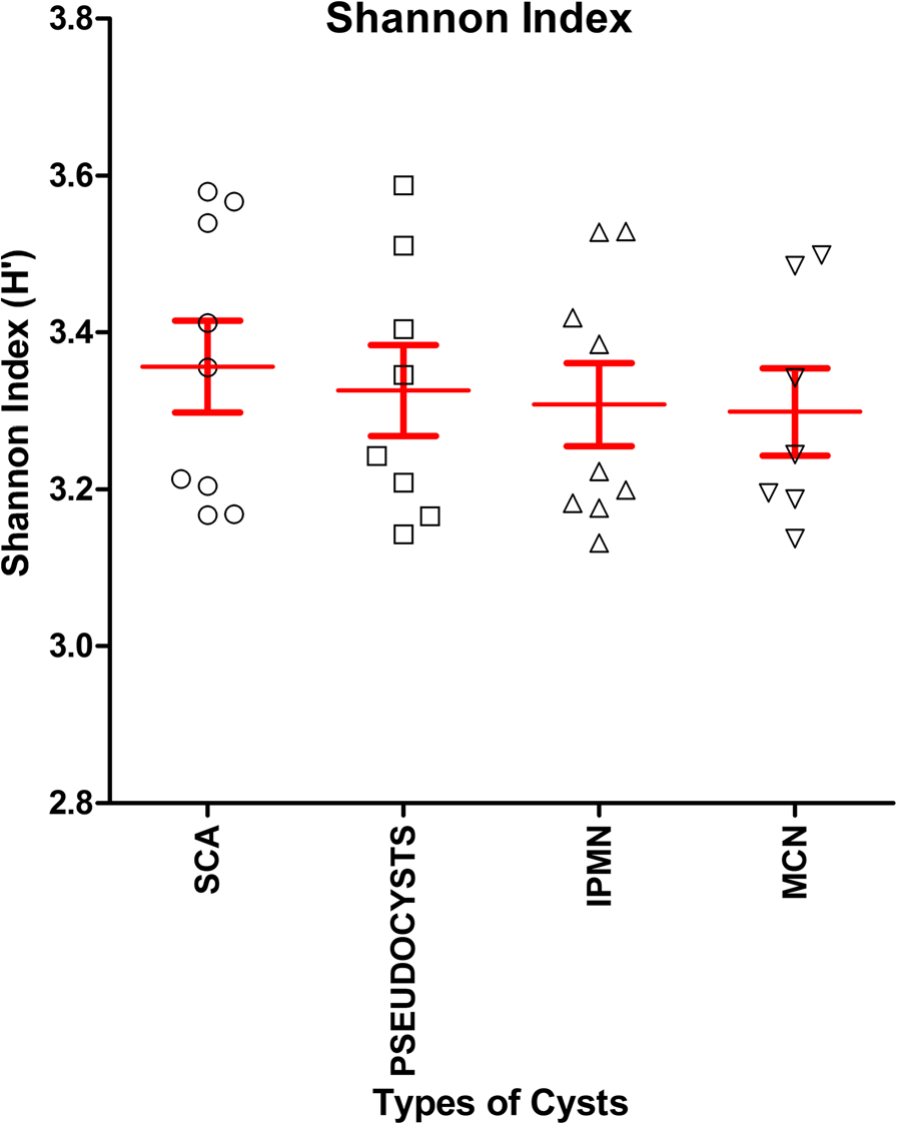
Shannon index for ecological microbial diversity in pancreatic cyst fluids. Employing the NGS results from PCF-derived DNA, the Shannon index for ecological diversity was calculated. There are no significant differences in the ecological diversity of the microbiome in the different types of PCF

To confirm the presence of these bacteria and gain more insight into the bacterial composition of the PCF, DNA obtained from the 23 fluids containing the highest apparent concentration of 16S rRNA copies were sent out for 16S rRNA de novo sequencing of V3-V4 region, using universal 16S rRNA primers which should allow identification of the bacteria present in such fluid at least on genus level. The results (shown in Fig. 2 and Additional file 2) are consistent with the presence of diverse bacterial ecosystems in such fluids, with as most predominant genera present *Bacteroides*, *Escherichia/Shigella*, and *Acidaminococcus*, but in total, 408 different genera were detected in the 33 samples analyzed, of which 93 genera were found in at least 50% of PCF samples analyzed. Different types of cysts were not statistically different with respect to microbial composition: for none of the 408 genera, a Bonferroni-corrected statistically significant difference in abundance was detected when serous cystadenoma, pseudocysts, IPMN, or mucous cystic neoplasm-derived fluids were compared. Furthermore, when low *P* values (Bonferroni-uncorrected *P* value < 0.05 but > 0.0001) were considered, it appeared that such findings were limited to very low abundant organisms (< 0.05% of all bacteria) that were relative low in one of the groups compared, more indicative of technical detection problems rather than a reflection of true biological differences between the groups (Additional file 2). In apparent agreement with the notion that the ecological niche provided by cyst fluid is relatively similar between different cyst manifestations is also the observation that the Shannon index for ecological diversity (*H′*) (Fig. 3) is not different between the different groups (*P* value = 0.99; one-way ANOVA).

### Diversity within the cyst type and resections

There were no significant difference in the diversity of bacterial microbiota seen in the 33 sample group of deep sequenced data, between the cyst types, or between the resected vs non resected groups or between the CA19.9 or CEA range-based classification groups. The results were in accordance with the bacterial presence in the different types of cysts based on the universal primer PCR sequencing.

### Microbial composition of pancreatic cyst fluid suggests the presence of a unique ecosystem

**Fig. 4:**
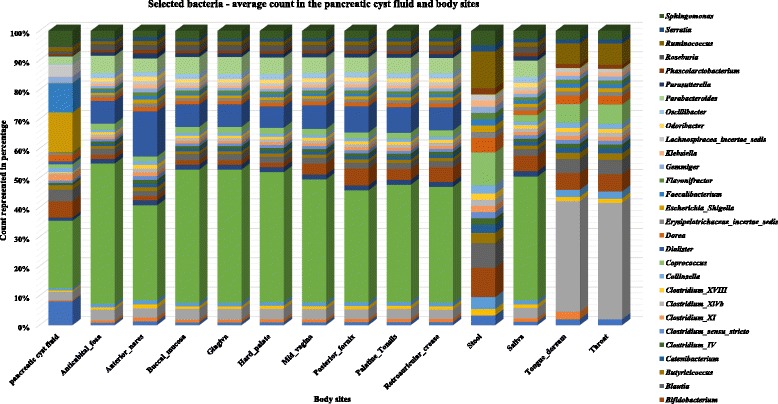
Pancreatic cyst microbiome is unique among other human body microbiomes. Publicly accessible databases were mined for composition of microbiomes at different human body sites and compared to those observed in PCF. It appears that there are 27 to 314 bacterial genotypes differently present in the PCF when compared to the selected body sites when analyzed via pairwise binomial test with high abundance PCF bacteria(*P* < 0.0001) and ANOVA test, respectively

**Fig. 5:**
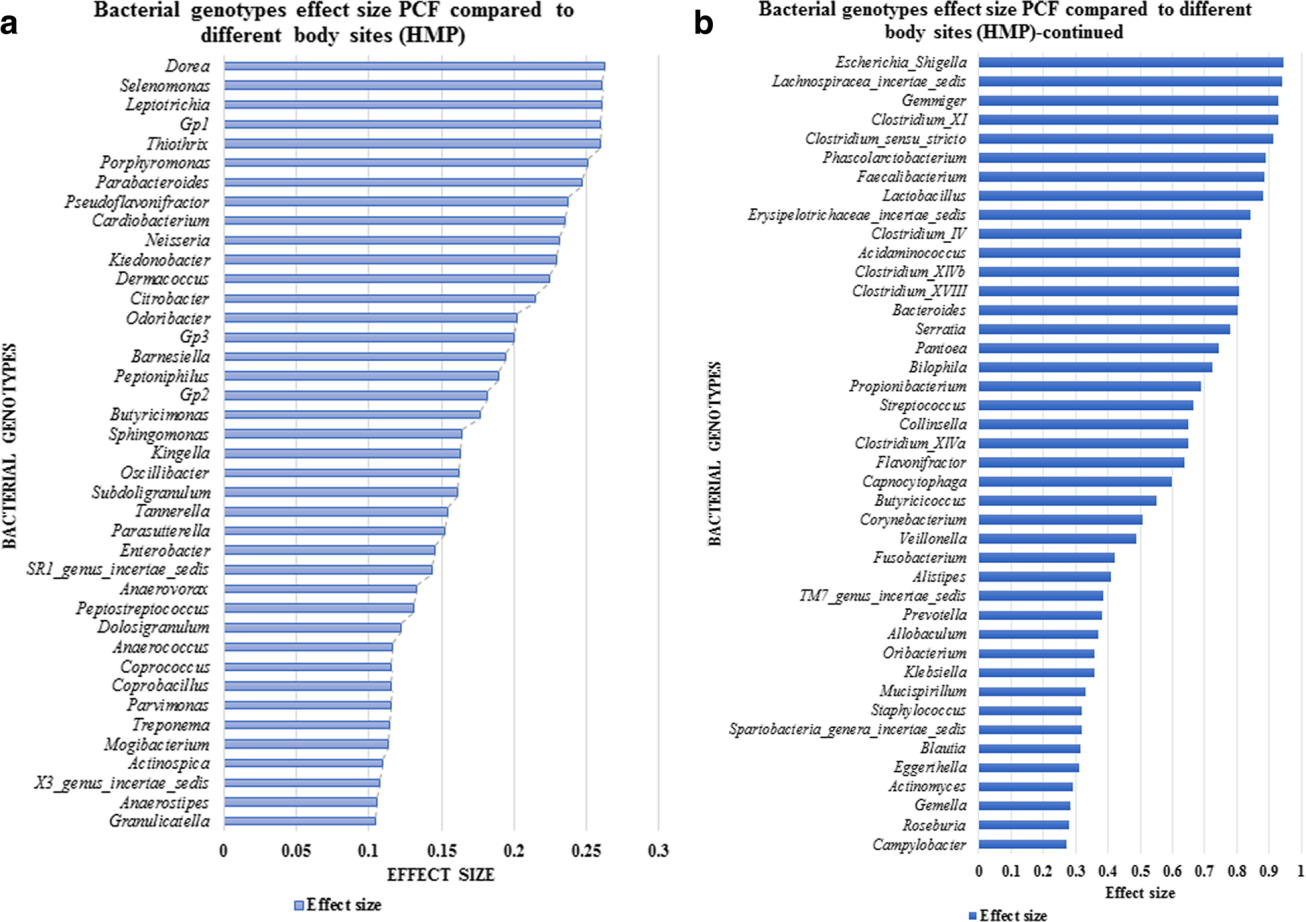
**a**, **b** Contribution of individual bacterial genera to the unique aspect of PCF fluid. For 82 different genera the relative in abundance in PCF was compared to that 13 other body sites and the relative contribution to the PCF-specific nature of the microbiome spectrum was calculated. In additional files information on a further 232 genera can be found. The results show that PCF contains a microbiome that is characterized by an overall uniqueness that cannot be attributed to a single genus

**Fig. 6:**
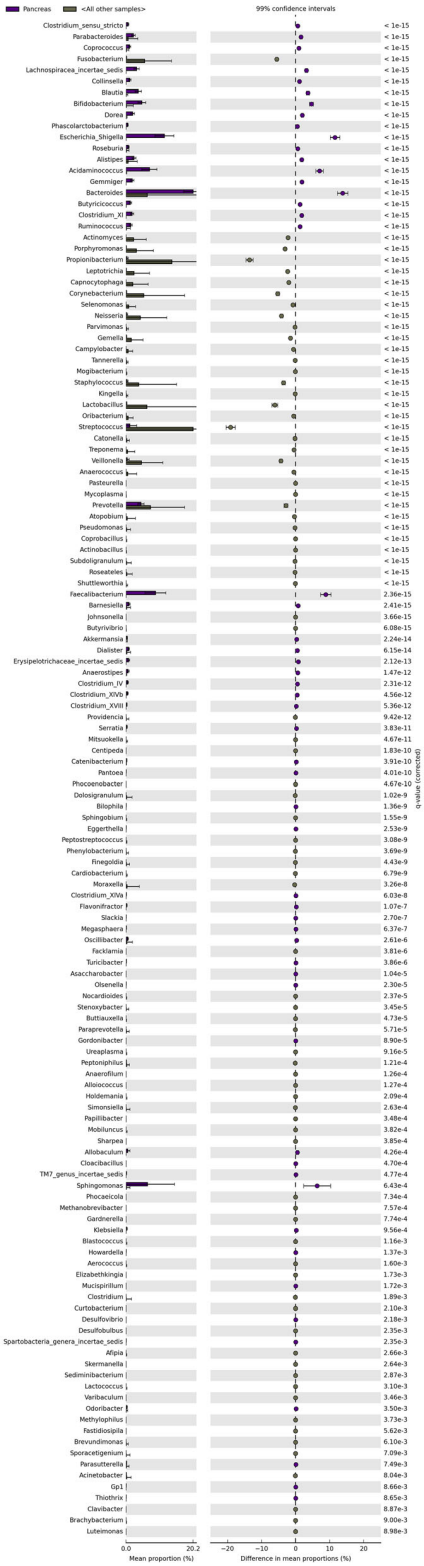
Bacterial commensals of PCF and selected different body sites comparison (HMP database) shows the difference in the distribution uniqueness in the PCF than the body sites. One hundred thirty-six bacterial genus of high and low abundance with *P* < 0.0001, and their difference of mean is plotted with 99% confidence interval, obtained via the Welch’s *t* test comparison between the groups

A comparison of 13 different body sites (Human Microbiome Project) with the PCF bacterial population showed 26 unique bacterial genotypes in PCF when compared with stool, 27 genotypes compared with throat, 28 genotypes compared with tongue dorsum, and 36 unique bacterial genotypes in the PCF which are not present within any of the other body sites selected (Fig. 4 and Additional file 4). ANOVA analysis, which is performed to identify the effect size of the bacterial population, reveals that 314 bacterial microbiota show an effect size variation between the bacterial commensals in PCF versus selected body sites, high effect size (0.1 to 0.9 and *P* < 0.01) bacterial populations are shown in Fig. 5, and total effect size variation is shown in Additional file 5. The PCA of different body sites and PCF are shown in Additional file 6: Figure S1. The Welch’s *t* test was performed to identify the specific bacterial populations having high and low abundance in the PCF when compared to HMP selected body sites. A total of 136 bacteria (Additional file 6: Figure S2 and Additional file 7) are identified, of which 17 abundant bacteria in PCF were highly unique and potentially pro-cancerous. They are, in ascending order of abundance in PCF, *Coprococcus* spp., *Collinsella* spp., *Butyricicoccus* spp., *Ruminococcus* spp., *Parabacteroides* spp., *Alistipes* spp., *Clostridium XI* spp., *Gemmiger* spp., *Dorea* spp., *Lachnospiracea incertae sedis* spp., *Blautia* spp., *Bifidobacterium* spp., *Sphingomonas* spp., *Acidaminococcus* spp., *Faecalibacterium* spp., *Escherichia/Shigella*, *and Bacteroides* spp. (*P* < 0.0001). A role for many of these bacteria in initiation and progression of colon, lung, and liver cancer has been suggested [26]. The following 15 bacterial genera were higher in all the 13 body sites used in this analysis: *Streptococcus* spp., *Propionibacterium* spp., *Lactobacillus* spp., *Fusobacterium* spp., *Corynebacterium* spp., *Veillonella* spp., *Neisseria* spp., *Staphylococcus* spp., *Porphyromonas* spp., *Prevotella* spp., *Leptotrichia* spp., *Actinomyces* spp., *Capnocytophaga* spp., *Gemella* spp., and *Selenomonas* spp. (*P* < 0.0001). These were also the least abundant bacteria present in the PCF*.* This comparison suggests that the PCF fluid bacterial colonization is unique and characterized by high abundant genera which have a tendency to propagate in the cancerous microenvironment, feeding the tumor (Fig. 6).

## Discussion

In the current study, we have demonstrated that 92.8% of a large and clinically representative collection of pancreatic cyst fluids (PCF) is host to a complicated ecosystem of bacteria. The distinct bacterial community had a rich species diversity which is very different from that observed elsewhere in the proximal human digestive tract. Although the EUS-FNA is not a sterile procedure, 7.8% of the PCF samples were negative for bacterial DNA arguing that the procedure did not have a major impact on our cultivation-independent bacteriological findings. Furthermore, the ecosystem observed by deep sequencing of pancreatic cyst material is inconsistent with that expected from contamination by the oral, nasal, pharyngeal, oesophageal, gastric, or duodenal flora. Members of the genus *Streptococcus* constitute by far the major component of the bacterial ecosystem in the esophagus [27], but while streptococcus was detected in all samples sequenced, it constitutes less than 1% of the total microbiota in any sample in our series, with the exception of one sample obtained from an IPMN. Likewise, the stomach is relatively sterile and dominated by *H. pylori*. The genus *Helicobacter*, however, was rare in our analysis, this genus the 67th genus in our ranking of our samples. Other bacteria found regularly in the stomach include *Streptococcus*, *Neisseria*, and *Lactobacillus*, and these species are not dominant in our analysis (being ranked as the 18th, 129th, and 54th most prevalent genus) [20]. Thus, the esophagus and stomach are an unlikely source of major contamination in our results. The duodenum can be home to substantial bacterial content, as also evident from conditions such as small intestinal bacterial overgrowth or SIBO. Valeria D’Argenio et al. recently reported on the bacterial composition of the duodenum in a cohort including 15 healthy individuals [28]. The most prevalent genus emerging from this analysis was *Propionibacterium*, which, although fairly often detected, was relatively rare in our analysis of pancreatic cyst fluids, with its abundance not even approaching 1% of the entire flora in any of the samples tested. Other genera dominating the duodenal microbiota include *Porphyromonas* (ranked 76th in cyst fluid), *Streptococcus*, *Neisseria*, and *Heamophillus* (ranked 21st, 58th, and 57th in cyst fluid)*.* Hence, the bacteria we observed in cyst fluid widely diverges from that expected if duodenal contamination was a major factor. Indeed in an analysis of microbiome of the healthy proximal tractus in our own institution (manuscript in preparation), we observed that *Streptococcus*, *Veillonella*, *Prevotella*, and *Pseudomonas* have the highest relative abundance from distal esophagus to the jejunum and proximal ileum. This situation is markedly different from that observed in pancreatic cyst fluids (although *Prevotella* was the 7th most abundant genus in pancreatic cyst fluid, this is still markedly lower as that observed in the proximal gastrointestinal tract of volunteers). In apparent agreement, in a preliminary series of experiments employing fluorescent in situ hybridization on surgically obtained pancreatic cyst material, bacteria were apparent (not shown). Thus, the most straightforward interpretation of results is that the pancreatic cyst is home to a previously unsuspected and also unique bacterial ecosystem.

The presence of such a bacterial ecosystem in pancreatic cysts raises important questions as to the role of the bacteria present in such cysts in the development of such structures. In general bacteria have been linked to transformation and trans-differentiation of endodermal epithelia, with *H. pylori* being the most important example. The genus *Helicobacter* was only marginally detected in pancreatic cyst fluid; other bacterial species linked to transformation in the intestine were, however, more prevalent. In particular, *F. nucleatum*, which is relatively predominant in a fraction of the samples and has been convincingly linked to neoplasm formation in the colon, excites interest in this respect. Emerging evidence suggests that *Fusobacterium* species detected in pancreatic cancer tissues is associated with a worse clinical outcome in pancreatic cancer patients [29]. But also other bacteria seen in pancreatic cyst fluids, even if it is at low abundance, may have a role here. Using NGS technology, multiple studies have compared oral microbiota between healthy individuals and those with pancreatic cancer [4, 30]. Although no correlation between known pro-oncogenic oral pathogens and pancreatic cancer was detected, Lin et al. showed that pancreatic cancer patients had significantly higher levels of *Bacteroides* genus compared to control subjects [30], which is in agreement with our finding that *Bacteroides* spp. were the most predominant genus in PCF. In addition, a recent pilot study using 16S rRNA gene sequencing on saliva specimens showed that while there was no difference in diversity of oral microbiota between patients with pancreatic ductal adenocarcinoma (PDAC), IMPN, or healthy controls, pancreatic cancer patients had higher levels of members of the phylum *Firmicutes* while healthy individuals were associated with higher relative abundances of *Proteobacteria* [31]. Evidence gathered from those studies supposes that pancreatic malignancy may be associated with changes in abundances of some groups of bacteria in the human digestive tract. Therefore, dysbiotic microbiota in the upper digestive tract including oral cavity may interrupt the unique ecosystem in pancreatic cyst fluids along the neoplastic process in pancreas. At bay with the notion of a causative role for the bacterial flora in pancreatic cyst formation, however, is the observation that both detection of bacteria per se or the composition of the cyst flora did not show correlation to clinical parameters and thus the role of the flora in cystogenesis, if any, awaits further study.

Pancreatic infections mainly arise from translocation of bacteria from the small bowel, and rarely from the colon and oropharyngeal route as demonstrated by study on *Veillonella* and *Bifidobacterium* spp. which were identified in pancreatic abscesses [32]. A study of Brook et al. has identified 158 bacterial species from pancreatic abscesses, of which 77 isolates were aerobic and the remaining 81 were anaerobic bacteria [33]. The most commonly detected microorganisms in infected pancreatic pseudocysts include often not only opportunistic bacteria like *E. coli*, *Enterobacter* spp., *Klebsiella* spp., and *Staphylococcus* spp. but also fungal isolates including *Candida albicans* (15 case studies) [34]. Importantly, EUS FNAB procedure caused serious *Clostridium perfringens* infections in five patients leading to pancreatitis and pancreatic cyst formation, which required surgical interventions [35]. Such studies illustrate the nature of the bacterial transfer from the early to mid-gut commensal bacteria to the pancreas. Yet none of these earlier studies have directly proved the presence of bacteria in the pancreatic cyst and its fluid. Its apparent divergence from the flora in the duodenum is probably a reflection of the specific conditions in pancreatic cysts which include an absence of contact with the digestive nutrient stream, an exposure to high levels of pancreatic secretes and the presence of abundant mucus. At present, clinical behavior of pancreatic cysts is very difficult to predict and adequate management of cysts represents one of the largest challenges in clinical gastroenterology. It is tempting to speculate, however, that changing conditions in cysts related to transformation of the structure to full-blown malignant cancer would also influence the *milieu interieur* formed by cyst fluid and hence have a profound effect on the bacterial composition. As such, changes in the bacterial communities may serve to detect such transformation in cyst-forming structures and may become useful for guiding clinical management.

Irrespective, however, of its potential as future diagnostic and prognostic marker, the present study shows an as yet unknown bacterial ecosystem in pancreatic cyst fluid. As it is evident that bacteria influence physiology and pathophysiology of their interacting epithelia everywhere in the gut, it is well possible that such interactions also exist in the pancreas and its cysts, and that the biology of the pancreatic cysts is in important ways shaped by this ecosystem. Studies addressing this possibility are currently in progress.

## Conclusions

The study reveals previously undescribed bacterial diversity present in human pancreas and its cyst fluids. As specific bacteria are associated with this body site, we propose that such bacteria may carry the potential to influence the development of pathophysiological processes in the pancreas. The study points out to the need to further explore the microbiome in this specific niche for diagnostic and therapeutic purposes.

## Additional files

Additional file 1: Detailed patient characteristics, samples characteristic of samples taken for NGS, gene accession numbers of Sanger sequenced samples and SRA numbers for the NGS selected samples, NGS sequenced V3-V4 variable regions of 16S rRNA. (XLSX 26 kb)

Additional file 2: Detailed quality of reads, taxonomic profiles, taxonomic distribution and taxonomic distribution rank. (XLSX 432 kb)

Additional file 3: SRA numbers of Human Microbiome Project used for comparisons. (XLSX 93 kb)

Additional file 4: Selected Bacterial counts of the PCF and selected body sites used for the binomial pairwise comparison. (XLSX 69 kb)

Additional file 5: ANOVA analysis statistics table for the PCF and selected body sites of bacterial distribution. (XLSX 89 kb)

Additional file 6: Figure S1. PCA of pancreatic cyst fluid (PCF) and 13 body site microbiome comparisons. PCA showing the difference between pancreatic cyst fluid and 13 different body site microbiome selected from Human Microbiome Project database. When compared 136 bacterial genus with *p* < 0.01 showing high (54) and low (82) abundance distribution between the PCF and 13 body site selected. This image constitutes the comparison between the PCF and 13 body site microbiomes (principal component analysis), A. PCA of antecubital fosa and pancreatic cyst fluids microbiome, B. PCA of anterior_nares and pancreatic cyst fluids microbiome, C. PCA of buccal_mucosa and and pancreatic cyst fluids microbiome, D. PCA of gingiva and pancreatic cyst fluids microbiome, E. PCA of hard_palate and pancreatic cyst fluids microbiome, F. PCA of mid_vagina and pancreatic cyst fluids microbiome, G. PCA of posterior_fornix and pancreatic cyst fluids microbiome, H. PCA of palatine_tonsils and pancreatic cyst fluids microbiome, I. PCA of retroauricular_crease and pancreatic cyst fluids microbiome, J. PCA of stool and pancreatic cyst fluids microbiome, K. PCA of saliva and pancreatic cyst fluids microbiome, L. PCA of tongue_dorsum and pancreatic cyst fluids microbiome and M. PCA of throat and pancreatic cyst fluids microbiome. Figure S2. Difference of mean of selected high and low abundance bacterial microbiome in PCF and 13 body sites together, respectively. Difference of mean between the bacterial genus distribution of pancreatic cyst fluid and 13 different body site microbiome selected from Human Microbiome Project database. When compared 17 bacterial genus (with *p* < 0.01) showing high abundance in PCF and 15 bacterial genus (with *p* < 0.01) showing high abundance in 13 body sites selected. (PPTX 7223 kb)

Additional file 7: Welch’s *t* test statistics run for the bacterial genotypes comparison calculation of the PCF and 13 different body sites microbiota. (XLSX 9711 kb)
