# Supplementary material for: Pancreatic cyst fluid harbors a unique microbiome
Source: Microbiome. 2017 Nov 9;5:147. doi: 10.1186/s40168-017-0363-6 (PMC5680603; doi:10.1186/s40168-017-0363-6)
Supplement: Supplementary file 6 — PCA of pancreatic cyst fluid (PCF) and 13 body site microbiome comparisons. PCA showing the difference between pancreatic cyst fluid and 13 different body site microbiome selected from Human Microbiome Project database. When compared 136 bacterial genus with p < 0.01 showing high (54) and low (82) abundance distribution between the PCF and 13 body site selected. This image constitutes the comparison between the PCF and 13 body site microbiomes (principal component analysis), A. PCA of antecubital fosa and pancreatic cyst fluids microbiome, B. PCA of anterior_nares and pancreatic cyst fluids microbiome, C. PCA of buccal_mucosa and and pancreatic cyst fluids microbiome, D. PCA of gingiva and pancreatic cyst fluids microbiome, E. PCA of hard_palate and pancreatic cyst fluids microbiome, F. PCA of mid_vagina and pancreatic cyst fluids microbiome, G. PCA of posterior_fornix and pancreatic cyst fluids microbiome, H. PCA of palatine_tonsils and pancreatic cyst fluids microbiome, I. PCA of retroauricular_crease and pancreatic cyst fluids microbiome, J. PCA of stool and pancreatic cyst fluids microbiome, K. PCA of saliva and pancreatic cyst fluids microbiome, L. PCA of tongue_dorsum and pancreatic cyst fluids microbiome and M. PCA of throat and pancreatic cyst fluids microbiome. Figure S2. Difference of mean of selected high and low abundance bacterial microbiome in PCF and 13 body sites together, respectively. Difference of mean between the bacterial genus distribution of pancreatic cyst fluid and 13 different body site microbiome selected from Human Microbiome Project database. When compared 17 bacterial genus (with p < 0.01) showing high abundance in PCF and 15 bacterial genus (with p < 0.01) showing high abundance in 13 body sites selected. (PPTX 7223 kb) [file 40168_2017_363_MOESM6_ESM.pptx]

## Slide 1
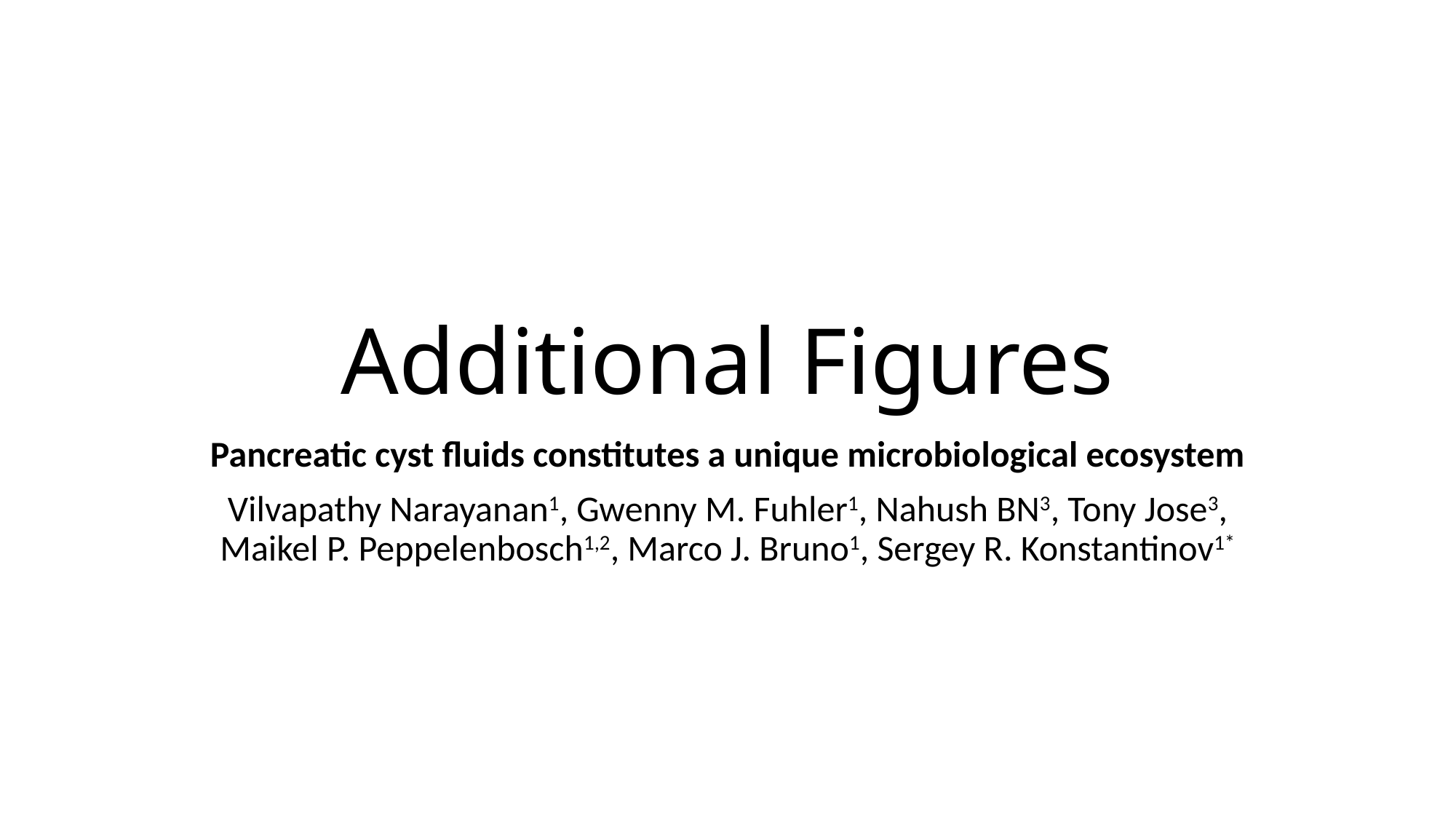

# Additional Figures
Pancreatic cyst fluids constitutes a unique microbiological ecosystem
Vilvapathy Narayanan1, Gwenny M. Fuhler1, Nahush BN3, Tony Jose3, Maikel P. Peppelenbosch1,2, Marco J. Bruno1, Sergey R. Konstantinov1*

## Slide 2
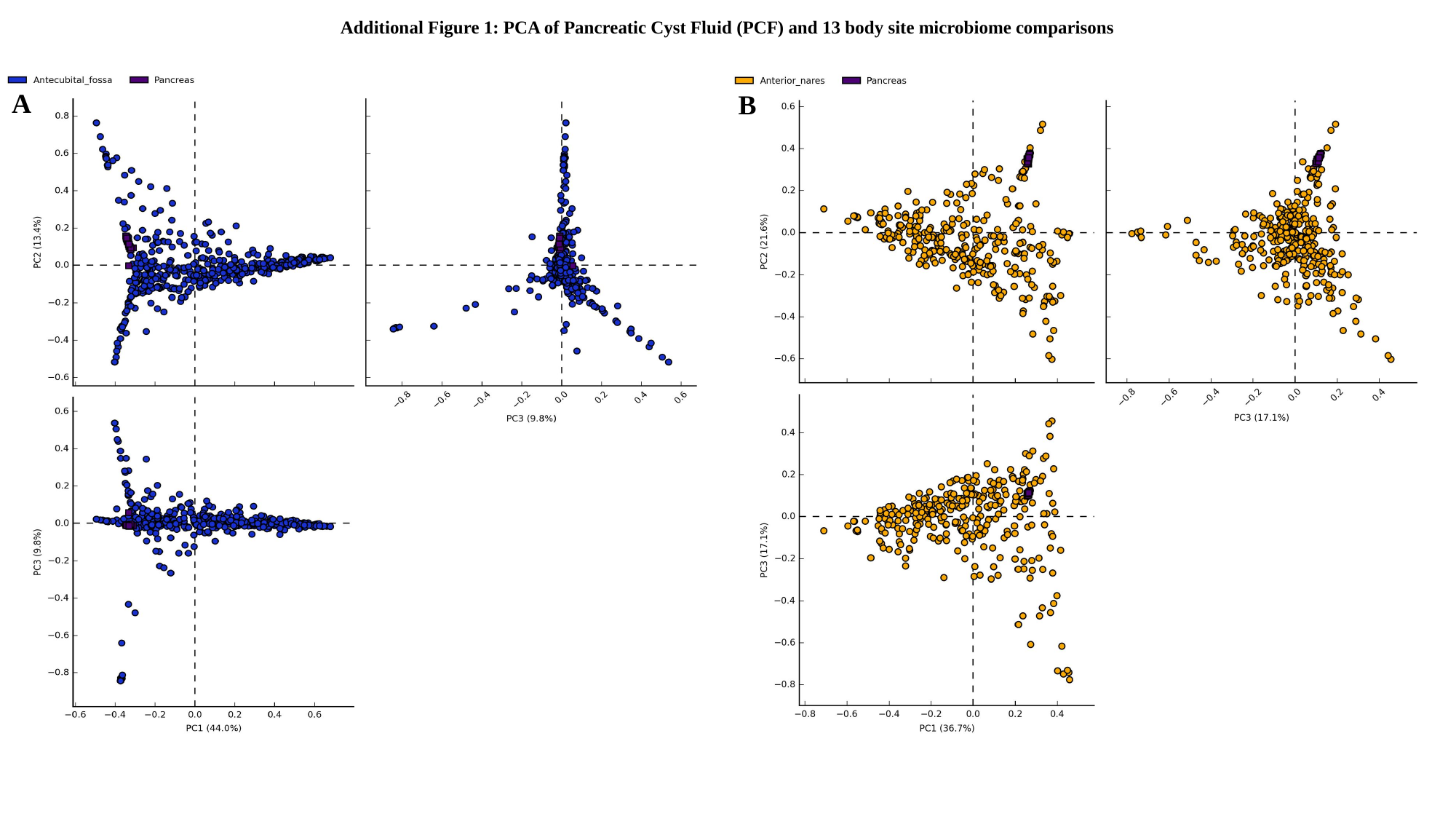

Additional Figure 1: PCA of Pancreatic Cyst Fluid (PCF) and 13 body site microbiome comparisons
A
B

## Slide 3
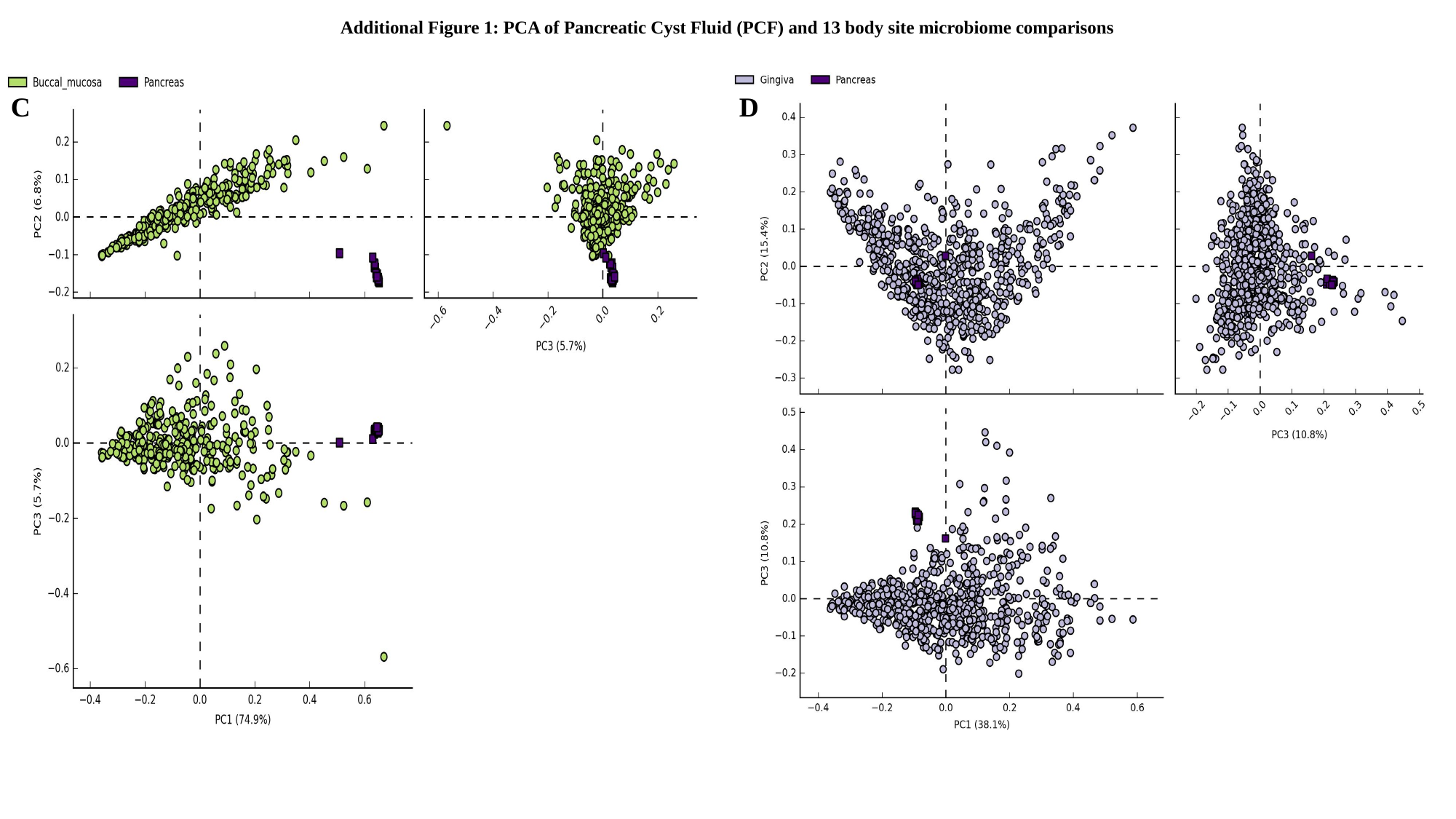

Additional Figure 1: PCA of Pancreatic Cyst Fluid (PCF) and 13 body site microbiome comparisons
C
D

## Slide 4
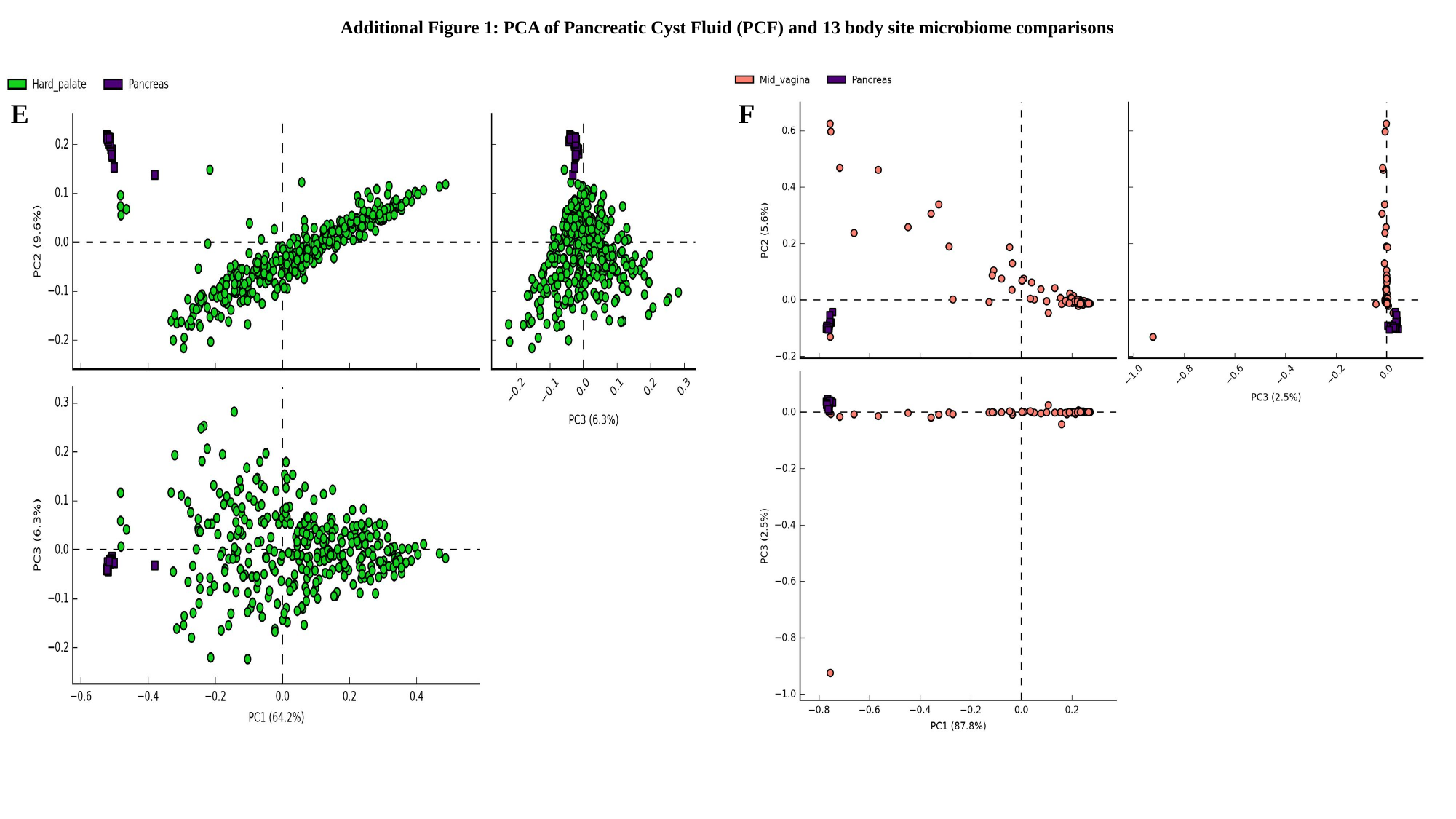

Additional Figure 1: PCA of Pancreatic Cyst Fluid (PCF) and 13 body site microbiome comparisons
E
F

## Slide 5
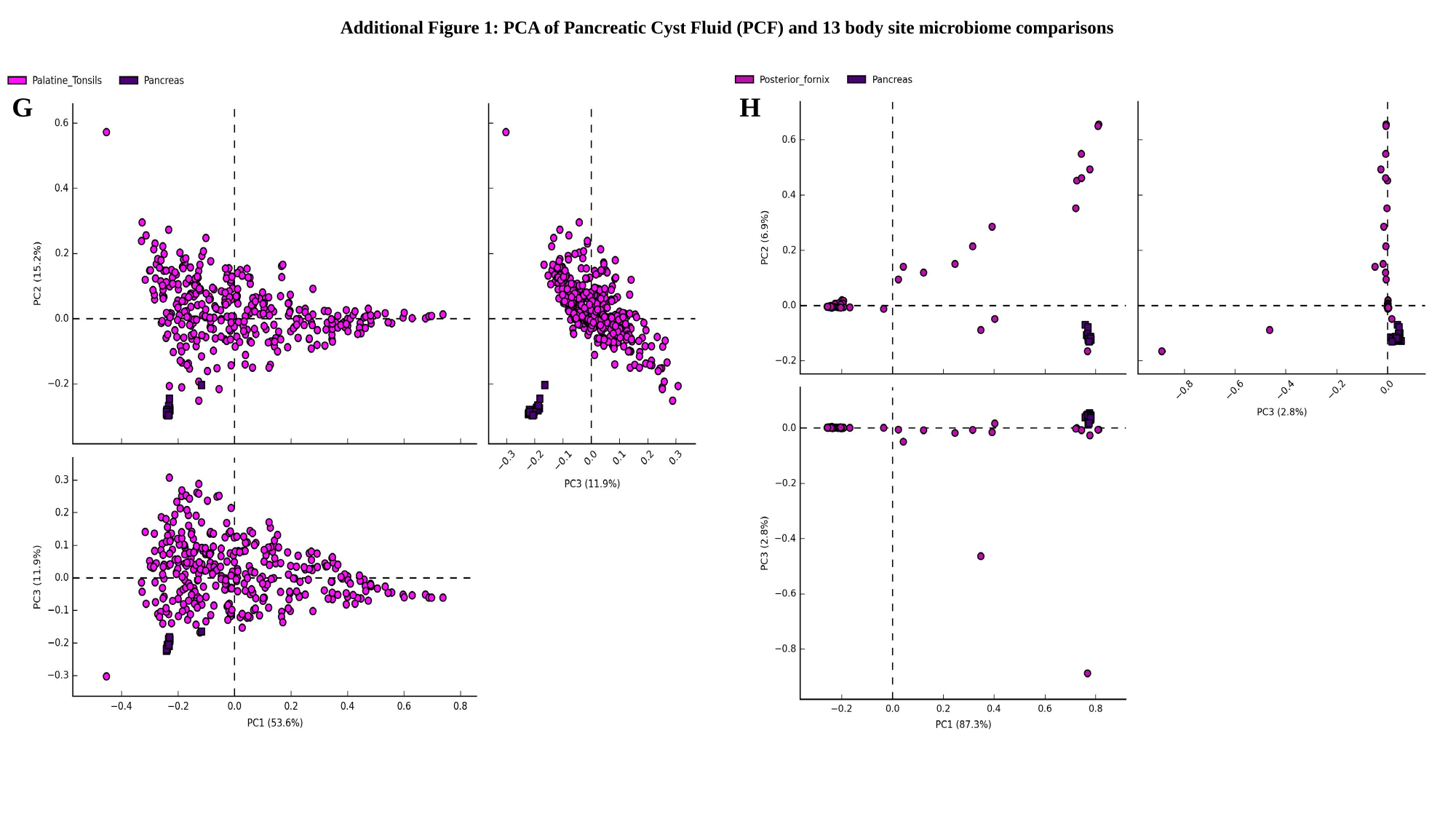

Additional Figure 1: PCA of Pancreatic Cyst Fluid (PCF) and 13 body site microbiome comparisons
G
H

## Slide 6
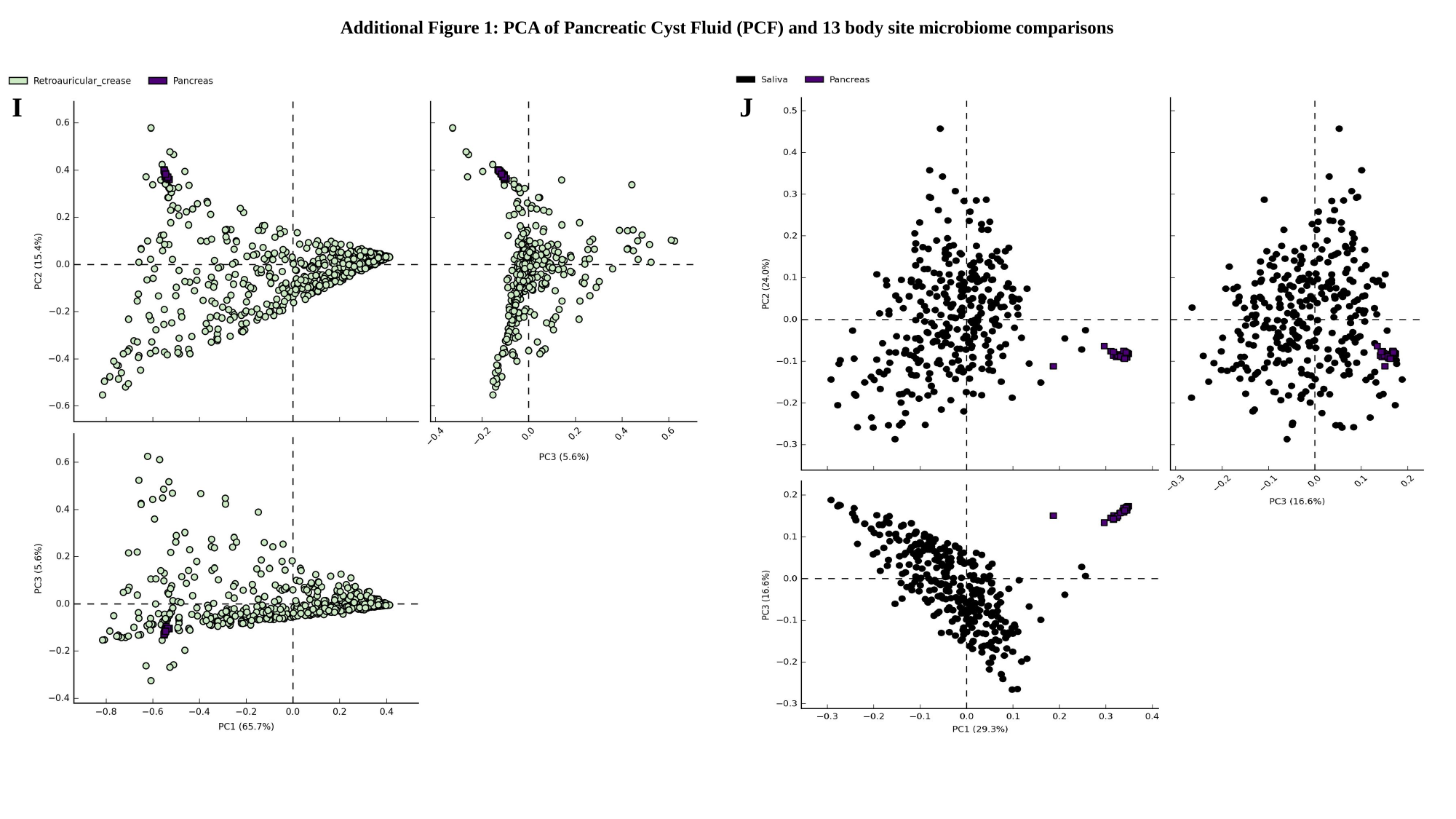

Additional Figure 1: PCA of Pancreatic Cyst Fluid (PCF) and 13 body site microbiome comparisons
I
J

## Slide 7
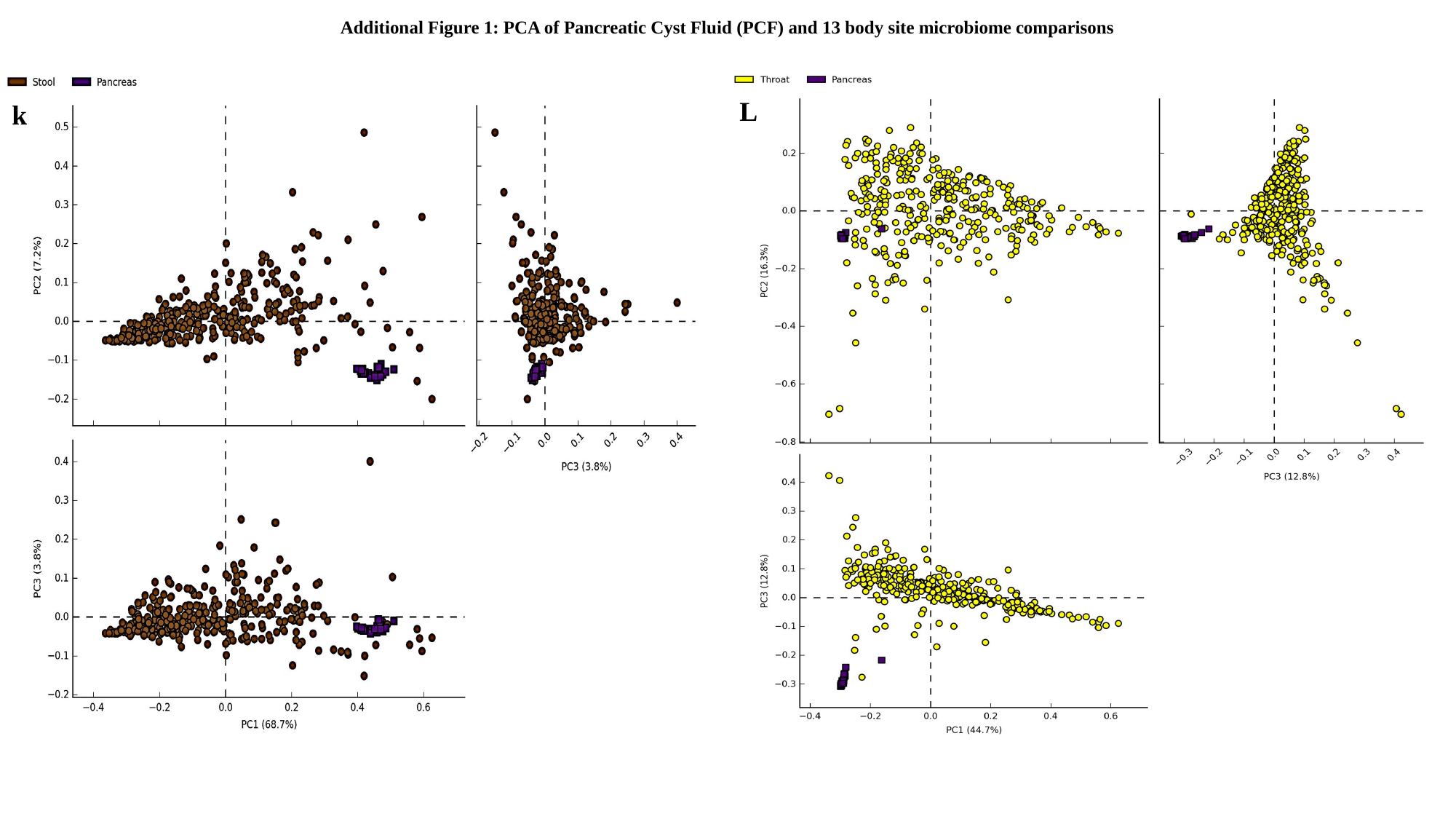

Additional Figure 1: PCA of Pancreatic Cyst Fluid (PCF) and 13 body site microbiome comparisons
L
k

## Slide 8
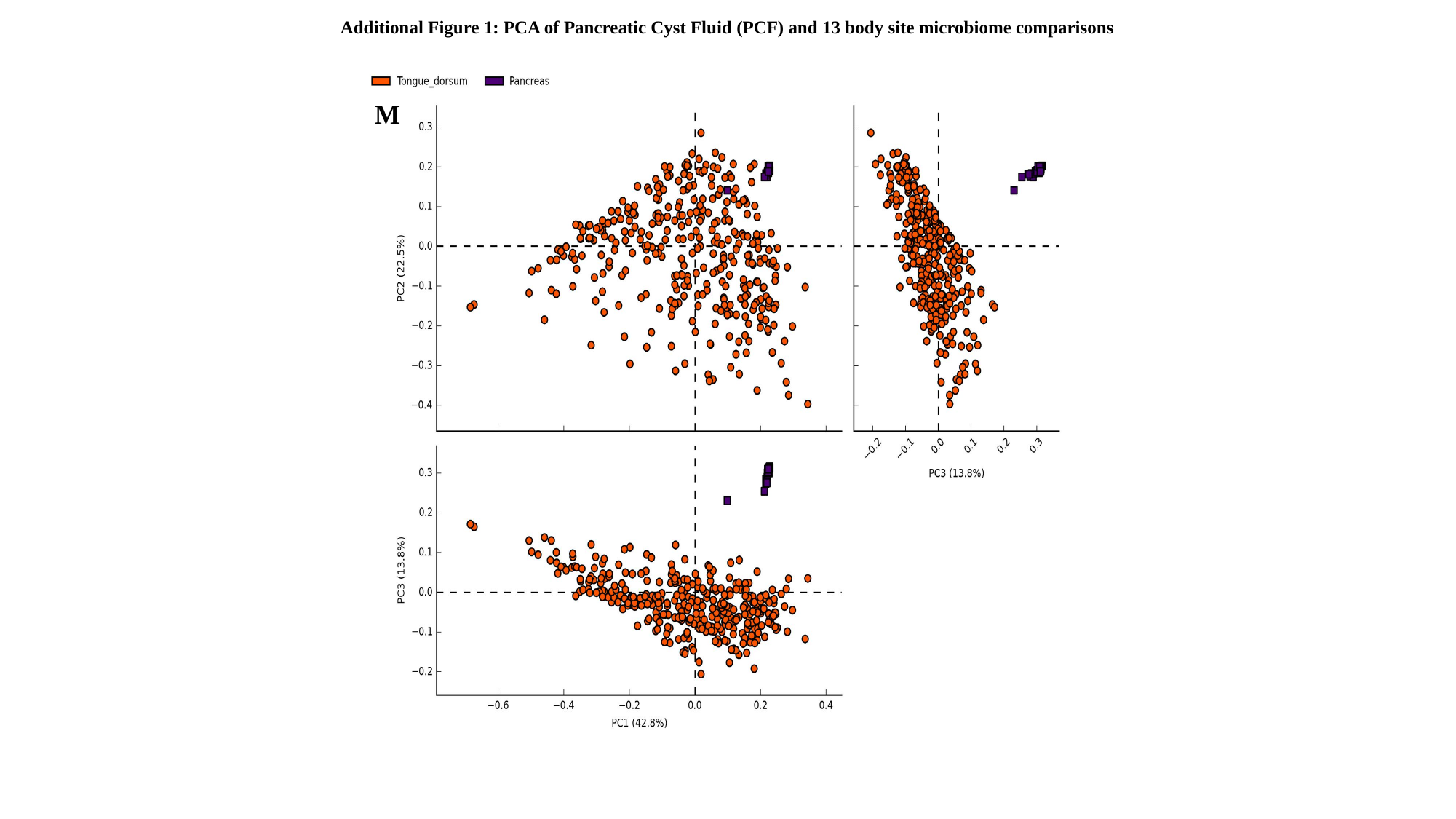

Additional Figure 1: PCA of Pancreatic Cyst Fluid (PCF) and 13 body site microbiome comparisons
M

## Slide 9
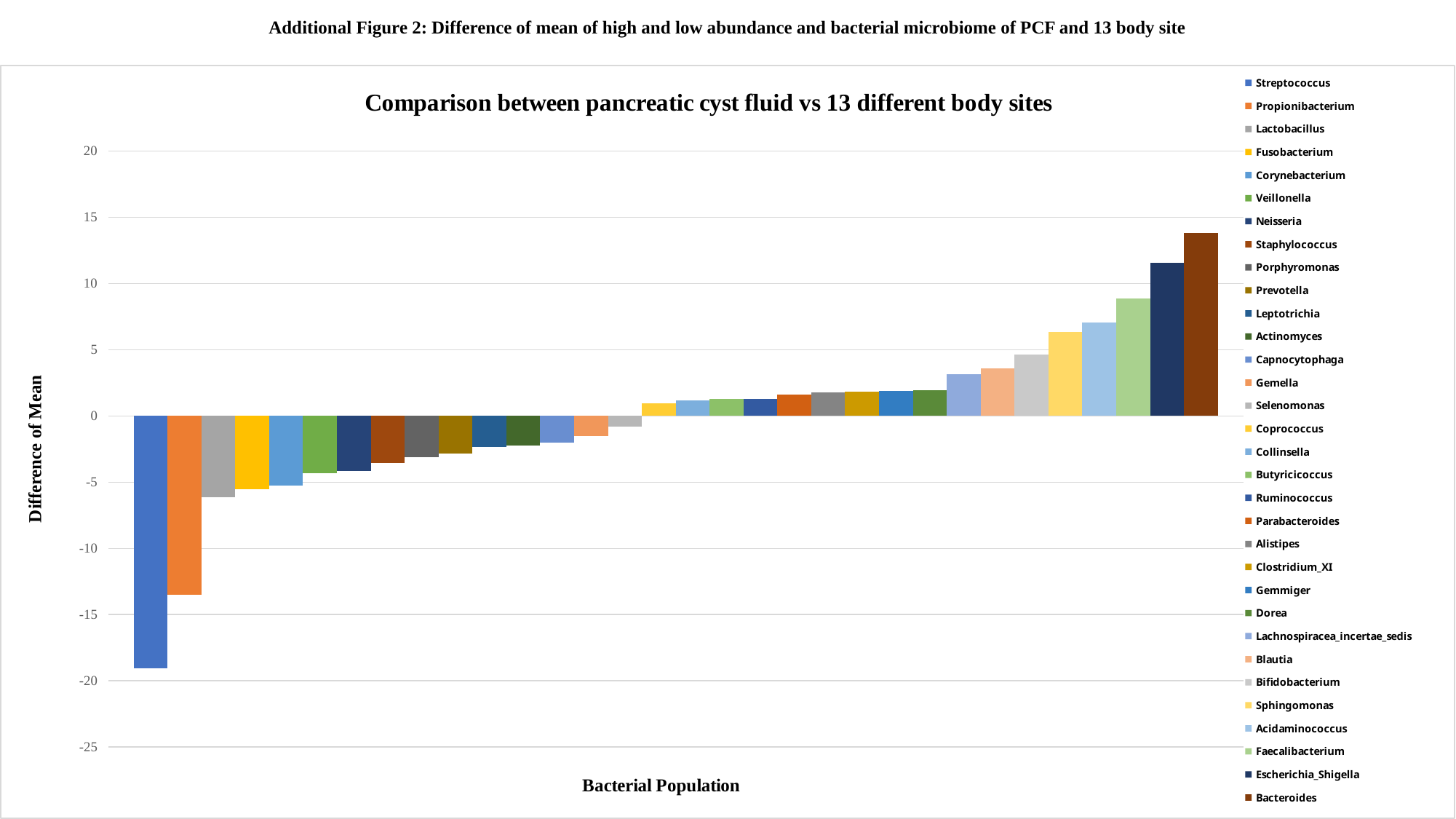

Additional Figure 2: Difference of mean of high and low abundance and bacterial microbiome of PCF and 13 body site
### Chart: Comparison between pancreatic cyst fluid vs 13 different body sites
| Category | Streptococcus | Propionibacterium | Lactobacillus | Fusobacterium | Corynebacterium | Veillonella | Neisseria | Staphylococcus | Porphyromonas | Prevotella | Leptotrichia | Actinomyces | Capnocytophaga | Gemella | Selenomonas | Coprococcus | Collinsella | Butyricicoccus | Ruminococcus | Parabacteroides | Alistipes | Clostridium_XI | Gemmiger | Dorea | Lachnospiracea_incertae_sedis | Blautia | Bifidobacterium | Sphingomonas | Acidaminococcus | Faecalibacterium | Escherichia_Shigella | Bacteroides |
|---|---|---|---|---|---|---|---|---|---|---|---|---|---|---|---|---|---|---|---|---|---|---|---|---|---|---|---|---|---|---|---|---|
| Difference between means | -19.0778581961 | -13.5103898829 | -6.11140421198 | -5.54741902178 | -5.23600706956 | -4.31469562238 | -4.13360372271 | -3.5352548072 | -3.08656002212 | -2.85871393657 | -2.34222223855 | -2.2348002582 | -2.03481329274 | -1.51011754855 | -0.808514562728 | 0.965385714552 | 1.15693522776 | 1.28546873751 | 1.30625763466 | 1.59720381868 | 1.80161890987 | 1.81136146022 | 1.86687656029 | 1.94695528469 | 3.16976795877 | 3.59074660675 | 4.65423068213 | 6.32821221757 | 7.0485671273 | 8.86218316226 | 11.5745139362 | 13.8055616603 |
